# Metastasis of Renal Cell Carcinoma to the Buccal Mucosa 19 Years after Radical Nephrectomy

**DOI:** 10.1155/2012/823042

**Published:** 2012-10-22

**Authors:** Hernani Gil-Julio, Fernando Vázquez-Alonso, Antonio J. Fernández-Sánchez, Ignacio Puche-Sanz, José F. Flores-Martín, José M. Cózar

**Affiliations:** Servicio de Urología, Hospital Universitario Virgen de las Nieves, 18012 Granada, Spain

## Abstract

Renal cell carcinoma (RCC) has high metastatic potential, which requires early diagnosis to optimize the chance of cure. Metastasis of RCC to the head and neck region is less common and metastasis to the buccal mucosa is extremely rare. This phenomenon occurs mostly in patients with generalized dissemination, especially with lung metastases. In this article we report a case of buccal mucosa metastasis from RCC in a 65-year-old man who presented 19 years after undergoing a left radical nephrectomy for clear cell RCC. Surgical excision of the buccal lesion was performed without evidence of recurrence or new metastatic lesions after 6 years of followup. To our knowledge, this is the first case of metastasis to the buccal mucosa from a RCC reported in the literature.

## 1. Introduction

Metastasis of RCC to the buccal mucosa represents an extremely rare finding and is regarded to have very poor prognosis. The metastasis of RCC to the head and neck region is relatively uncommon and can be found in 8–16% of all cases [1–5], usually associated with lesions in other sites. The rich vascular structure of RCCs facilitates hematogenous extension and the development of distant metastases. The most important hematogenous extension route in RCC is the vena cava system, which leads to the lung. Metastatic tumours to the buccalmucosa generally present with nonspecific symptoms and signs. Surgical excision is considered the first line of treatment and the decision should be based on the evidence of other organs involvement, the patient's general condition [2, 4, 6], and the use of radiotherapy [3, 5] or antiangiogenic therapy [7].

In this paper, we report a rare case of metastasis to the buccal mucosa from an RCC that occurred 19 years after left radical nephrectomy. 

## 2. Case Report

**Figure 1:**
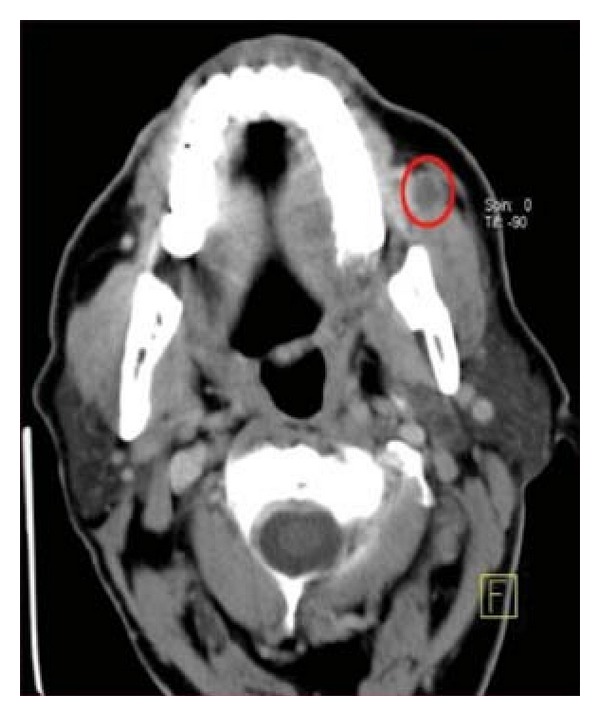
Head CT image showing a malignant lesion in the left buccal mucosa.

A65-year-old otherwise healthy male patient presented to clinic with a sensation of discomfort in his left cheek. His past history is significant for left radical nephrectomyperformed 19 years earlier for clear cell renal cellcarcinomawithrenalveinthrombosis (pT3aN0M0), and there were no other sites of disease evident upon current presentation. Computed Tomography scan detected a 10 mm × 8 mm mass suggestive of malignant lesion in theleftbuccalmucosa (Figure 1) which was then surgically removed. Pathological examination showed metastaticclear cell renal cell carcinoma with clear excision margins. After 6 years of clinical followup and serial radiological controls, the patient showed no signs of local recurrence or new metastatic lesions.

## 3. Discussion

RCC represents 3% of all adult malignancies [3, 5]. Male to female ratio is 1.5 : 1 and it is more frequently diagnosed during the fifth and sixth decades of life [5].

RCC is the most common malignancy of the kidney [3, 8] and represents approximately 90% of malignant renal tumours in adults [2]. Kidneys receive approximately 25% of the cardiac output therefore highly vascular renal tumours like RCC have a high metastatic potential [1]. The most common sites for RCC metastasis are the lungs, regional lymph nodes, bone, liver, contralateral kidney, brain, pancreas, and skin [1–5]. 

RCC is the third most frequent neoplasm reported to metastasise to the head and neck region, after breast and lung carcinoma [3–5]. Approximately1–3% ofpatients with RCC,without lesionsin other sites,have metastasis confined to the head and neck [2, 3, 5, 8].

The thyroid gland is the most common site in the head and neck region for RCC metastasis [1, 5]. Only 1% of all malignancies in the oral cavity are metastatic foci. Our case appears to be unique, no cases of metastases to the buccal mucosa havebeen reported before.

Almost 25% of patients with RCC have distant metastases at the time of diagnosis. This trend has remained stable over the last two decades despite advances in methods of detection, especially abdominal imaging techniques [8].

The tumour can metastasise after long periods of latency, with reports of up to 20 [3] or even up to 25 years [8] between the primary and metastatic presentation. Oral metastases of RCC are a manifestation of widespread disease [2, 3], especially when found with lung metastases [3], and imply a 5-year survival rate of 13–50% [2, 8], although a high proportion of patients develop further metastasis [8]. It is also well known that favourable prognosis is associated with solitary metastatic focus and longer interval between the primary treatments and the appearance of the metastasis [9].

RCC invades the local vascular network of the kidney, spreading through the systemic circulation [3]. If there are no signs of pulmonary disease spread may occur through Batson's venous plexus [3, 5], which extends from the skull to the sacrum or via the thoracic duct [3]. This valveless system offers little resistance to the spread of tumour emboli, especially during Valsalva maneuvers, during which there is an increase of intra-abdominal and intrathoracic pressure, allowing bypass of the pulmonary filters [3, 5]. It has also been postulated that renal cell carcinoma metastasis could arrive at the head and neck via normal hematogenous flow through the lungs, leading to microscopic seeding of the lung parenchyma, which would not be visible on routine chest radiographs [5, 6]. Another possibility is that a microscopic metastatic lesion might start to grow rapidly, with a consequent decrease in host immunopotency [6].

CT scan is the radiologic investigation of choice in assessing the extent of the metastatic lesion [5, 10]. Angiography will show a highly vascular mass [5]. Magnetic resonance scanning can also be helpful, especially in assessing residual disease after radiotherapy treatment [5, 10]. 

Management of these patients should be individualised based on the presence or absence of metastasis to different organs and the patient's general health [2, 4]. Surgical excision is recommended as the primary line of treatment especially for those with no other organ involvement [2, 4–6].

The goal of surgical treatment of buccal metastasis is usually palliative providing patients with comfort and pain relief and at the same time preventing bleeding and infections [2]. The role of radiotherapy as the primary approach is controversial and has been reported by some authors for palliative management [2]. Other authors suggest that some metastatic lesions respond well to higher doses of radiotherapy with good local control [5]. RCC is traditionally described as being radioresistant [5, 7] and a chemoresistant tumour, with the average response rate to chemotherapy being as low as 7% [2, 5]. Recent data regarding antiangiogenic therapy for metastatic RCC are encouraging [7]. 

In conclusion, the presenceof metastasis inoral mucosadue to RCC is rareand does not necessarily implypoorprognosisas has been describedfor metastases in thehead and neck. However, reviewingthe literature, wesuggest that the treatmentof metastaticRCC shouldbe individualised.Asurgical approach tailored tosolitary lesions, as in our case,may be beneficialfor controlling metastatic disease.
